# The Structure of Relationships between the Human Exposome and Cardiometabolic Health: The Million Veteran Program

**DOI:** 10.3390/nu13041364

**Published:** 2021-04-19

**Authors:** Kerry L. Ivey, Xuan-Mai T. Nguyen, Daniel Posner, Geraint B. Rogers, Deirdre K. Tobias, Rebecca Song, Yuk-Lam Ho, Ruifeng Li, Peter W. F. Wilson, Kelly Cho, John Michael Gaziano, Frank B. Hu, Walter C. Willett, Luc Djoussé

**Affiliations:** 1 Massachusetts Veterans Epidemiology and Research Information Center (MAVERIC), Boston Veterans Affairs Healthcare System, Boston, MA 02130, USA; xuan-mai.nguyen@va.gov (X.-M.T.N.); dcposner@bu.edu (D.P.); Rebecca.Song@va.gov (R.S.); Yuk-Lam.Ho@va.gov (Y.-L.H.); Kelly.Cho@va.gov (K.C.); michael.gaziano@va.gov (J.M.G.); ldjousse@rics.bwh.harvard.edu (L.D.); 2 South Australian Health and Medical Research Institute, Infection and Immunity Theme, Adelaide 5000, Australia; Geraint.Rogers@sahmri.com; 3 Department of Nutrition, Harvard T.H. Chan School of Public Health, Boston, MA 02115, USA; dtobias@bwh.harvard.edu (D.K.T.); rli@hsph.harvard.edu (R.L.); fhu@hsph.harvard.edu (F.B.H.); wwillett@hsph.harvard.edu (W.C.W.); 4 Division of Aging, Department of Medicine, Brigham and Women’s Hospital, Boston, MA 02115, USA; 5 Department of Medicine, Harvard Medical School, Boston, MA 02115, USA; 6 Division of Preventive Medicine, Department of Medicine, Brigham and Women’s Hospital, Boston, MA 02115, USA; 7 Department of Epidemiology, Boston University School of Public Health, Boston, MA 02118, USA; 8 Atlanta Veterans Affairs Medical Center, Decatur, GA 30033, USA; pwwilso@emory.edu; 9 Department of Medicine, Division of Cardiovascular Disease, Emory University Schools of Medicine and Public Health, Atlanta, GA 30322, USA; 10 Division of General Internal Medicine, Department of Medicine, Brigham and Women’s Hospital, Boston, MA 02115, USA; 11 Department of Epidemiology, Harvard T.H. Chan School of Public Health, Boston, MA 02115, USA; 12 Channing Division of Network Medicine, Department of Medicine, Brigham and Women’s Hospital, Harvard Medical School, Boston, MA 02115, USA

**Keywords:** exposome, diet, lifestyle, demographics, cardiovascular disease, cholesterol, triglycerides, blood pressure, glycemic control

## Abstract

The *exposome* represents the array of dietary, lifestyle, and demographic factors to which an individual is exposed. Individual components of the exposome, or groups of components, are recognized as influencing many aspects of human physiology, including cardiometabolic health. However, the influence of the whole exposome on health outcomes is poorly understood and may differ substantially from the sum of its individual components. As such, studies of the complete exposome are more biologically representative than fragmented models based on subsets of factors. This study aimed to model the system of relationships underlying the way in which the diet, lifestyle, and demographic components of the overall exposome shapes the cardiometabolic risk profile. The current study included 36,496 US Veterans enrolled in the VA Million Veteran Program (MVP) who had complete assessments of their diet, lifestyle, demography, and markers of cardiometabolic health, including serum lipids, blood pressure, and glycemic control. The cohort was randomly divided into training and validation datasets. In the training dataset, we conducted two separate exploratory factor analyses (EFA) to identify common factors among exposures (diet, demographics, and physical activity) and laboratory measures (lipids, blood pressure, and glycemic control), respectively. In the validation dataset, we used multiple normal regression to examine the combined effects of exposure factors on the clinical factors representing cardiometabolic health. The mean ± SD age of participants was 62.4 ± 13.4 years for both the training and validation datasets. The EFA revealed 19 Exposure Common Factors and 5 Physiology Common Factors that explained the observed (measured) data. Multivariate regression in the validation dataset revealed the structure of associations between the Exposure Common Factors and the Physiology Common Factors. For example, we found that the factor for fruit consumption was inversely associated with the factor summarizing total cholesterol and low-density lipoprotein cholesterol (LDLC, *p* = 0.008), and the latent construct describing light levels of physical activity was inversely associated with the blood pressure latent construct (*p* < 0.0001). We also found that a factor summarizing that participants who frequently consume whole milk are less likely to frequently consume skim milk, was positively associated with the latent constructs representing total cholesterol and LDLC as well as systolic and diastolic blood pressure (*p* = 0.0006 and <0.0001, respectively). Multiple multivariable-adjusted regression analyses of exposome factors allowed us to model the influence of the exposome as a whole. In this metadata-rich, prospective cohort of US Veterans, there was evidence of structural relationships between diet, lifestyle, and demographic exposures and subsequent markers of cardiometabolic health. This methodology could be applied to answer a variety of research questions about human health exposures that utilize electronic health record data and can accommodate continuous, ordinal, and binary data derived from questionnaires. Further work to explore the potential utility of including genetic risk scores and time-varying covariates is warranted.

## 1. Background

Cardiovascular disease (CVD) is the leading cause of adult mortality globally [1] Strategies aimed at reducing CVD rates involve modulation of markers of CVD risk. In particular, elevated circulating cholesterol and triglyceride levels are associated with higher CVD risk and mortality and are principal targets for risk reduction [2,3]. Further, elevated blood pressure is one of the leading non-communicable disease risk factors [4], and elevated glycated hemoglobin is also a predictor of cardiovascular disease [5,6,7,8]. As such, strategies aimed at improving lipid profile, blood pressure, and glycemic control are urgently needed.

The array of external factors an individual is exposed to, referred to as the exposome, represents a complex network of interrelationships within and between different components comprising diet, lifestyle, and demographics. Despite the well-documented ability of individual exposome components, or groups of exposome components, to influence many aspects of human physiology [9,10,11], surprisingly little is known about how the complex array of exposures as a whole shape the cardiometabolic risk profile. Reductionist approaches, such as single-exposure models, are unable to account for the complex interactions between the many potential exposome components and their effect on physiology [12]. The absence of models that integrate the many different components undermines the capacity of current studies of exposome components to draw robust generalizations. This project therefore aims to model the system of relationships underlying the way in which the exposome, as a whole, shapes the cardiometabolic risk profile. To achieve this aim, we utilized a truly unique dataset generated from an exposome assessment, as well as longitudinally assessed markers of cardiometabolic health in the Million Veteran Program.

## 2. Methods

### 2.1. Study Population

Between January 2011 and November 2019, approximately 800,000 Veterans enrolled in the Million Veteran Program (MVP) [13]. The current prospective study draws from the approximately 350,000 Veterans that had enrolled in the MVP between January 2011 and 2016. Of the 297,937 participants that had exposure data, 182,363 participants were excluded if they were using antilipemic, antihypertensive, and/or hypoglycemic medications during either the exposure-assessment or outcome-assessment periods (Supplementary Table S1). A further 79,078 participants were excluded as they had incomplete exposure and/or physiology data. Consequently, the final analysis included 36,496 MVP participants. Consent was obtained in accordance with all VA policies and under the authority of the VA Central IRB [13].

### 2.2. Exposome Assessment

Variables representing the exposome were observed (measured) using various questionnaires. At enrollment, participants completed a baseline questionnaire in which they reported their date of birth, height, smoking status, gender, ethnicity, and race. Participants also completed a lifestyle questionnaire in which they reported body weight, frequency of light, moderate, and vigorous physical activities both during and outside of work hours, smoking status, and number of hours per week spent in sedentary activities (watching television, DVDs, or videos, using a computer, or playing video games). Body mass index (BMI) was calculated as weight (kg)/height (m) [2].

A food frequency questionnaire (FFQ) was used to assess habitual dietary intake over the year preceding lifestyle questionnaire administration. Participants were asked to describe the average consumption frequency of 67 different foods. “For each food listed, please mark the column indicating how often, on average, you have used the amount specified during the past year”. Pre-specified answers were as follows: “Never or less than once a month; 1 to 3 per month; once a week; 2 to 4 per week; 5 to 6 per week; once (1) a day; 2 to 3 per day; 4 to 5 per day; or 6+ per day”.

For this study, we included 83 measured (observed) variables to represent the exposome. This is a large number of individual variables, many of which are not independent from one another due to patterns of behaviors amongst participants. In order to make sense of the numerous individual measured (observed) exposome variables, it was imperative that we reduce the dimensionality of the exposome variables to a smaller set of common factors representing groups of covarying measured (observed) variables. The methods to achieve this dimension reduction are detailed in the statistical analysis section.

### 2.3. Assessment of Physiological Markers of Cardiometabolic Health

A panel of clinicians reviewed lab measurements across all VA locations and adjudicated discrepancies to ensure lab measure consistency. Electronic medical records, adjudicated by two clinicians, were used to ascertain the markers of cardiometabolic health, which included systolic and diastolic blood pressures, as well as the concentration of total cholesterol, low-density lipoprotein cholesterol (LDLC), high density lipoprotein cholesterol (HDLC), triglycerides, HbA1c, and glucose.

This study included the physiological variables that were assessed during the 1–4 years post lifestyle questionnaire administration. For each individual marker of cardiometabolic health, analyses utilized the mean measurement of all of the assessments taken during this period, as well as the maximum recorded measurement during this time period. For each physiology parameter, measurements were excluded if they were taken within 4 days of a previous measurement [14], if they had negative values, or if they had values that were three interquartile ranges from the 25th and 75th percentiles. Data from casually obtained specimens were used in the analysis without regard to fasting status.

### 2.4. Assessment of Medication Usage

Electronic medical records were used to ascertain antilipemic agent usage during the 1 year preceding exposure assessment and during the outcome assessment period. Two clinicians adjudicated the list of antilipemic agents and included specific medications and doses from the following Generic Adjudication Classes: alirocumab; atorvastatin; atromid; bezafibrate; bezalip retard; cholestyramine; choloxin; clofibrate; colesevelam; colestipol; dextrothyroxine; evolocumab; ezetimibe; ezetimibe/simvastatin; fenofibrate; fenofibric acid; fish oil; gemfibrozil; icosapent ethyl; lomitapide; mevacor; mipomersen; niacinamide; omega-3; omega-3 acid; probucol; rosuvastatin; and statin. The same method of adjudication was used to ascertain usage of antihypertensive medications and combinations, as well as use of hypoglycemic agents.

### 2.5. Statistical Analysis

Before analysis, participants were randomly divided into one of two groups: a training dataset containing 66.6% of participants (*n* = 24,411) or a validation dataset containing the remaining 33.3% of participants (*n* = 12,085). See Supplementary Figure S1 for details.

The advent of multidimensional cohorts that both assess the exposome and are linked to electronic health records has resulted in large and complex datasets that comprise normally and non-normally distributed data that can be continuous, ordinal, categorical, and binary. There are many techniques available for reducing data dimensionality, which can be broadly categorized into supervised analyses (such as decision trees) and unsupervised analyses. Of the unsupervised analytic approaches, methods such as cluster analysis were not implemented as they would have reduced the number of observations (participants) by grouping them into a smaller set of clusters. Instead, we were aiming to achieve a reduction in the number of variables by grouping them into a smaller set of factors. We achieved this aim through implementation of common exploratory factor analysis. In fact, the use of common exploratory factor analysis in biomedical research is well-tested and effective [15,16], representing an established method whereby “hidden” relationships between the assumed latent variables and the initial observed (measured) variables can be uncovered [17]. To make sense of these data, this study aimed to (i) holistically examine the complex networks of interrelationships that define the exposome and clinical cardiometabolic risk profile; (ii) represent theoretical constructs that are unmeasurable or unmeasured; (iii) include parameter-specific measurement error; and (iv) integrate a number of techniques into one framework, accounting for the range of distributions, units, and relations within and between exposures and cardiometabolic health. Through applying tetrachoric and polychoric, common exploratory factor analysis followed by multivariable-adjusted regression analysis, our methods allowed us to observe the structure of relationships within and between the human exposome and subsequent markers of cardiometabolic health in this large, metadata-rich, prospective cohort of adult US Veterans.

The first stage of the analysis identified the latent constructs (common factors) that best described the shared covariance of the observed (measured) exposures and the physiological variables in the training dataset. These unobservable latent constructs are essentially hypothetical constructs that are used to represent groups of interconnected measured variables [18]. Exploratory factor analyses were used to evaluate the latent constructs and underlying structure because there were multiple hypotheses and extremely limited *a priori* knowledge of how observed (measured) variables might cluster, and because we aimed to develop a measurement model of latent variables, and not to merely identify a linear combination of variables, as is the case in principal component analysis.

For exposure variables, the exploratory factor analysis used tetrachoric and polychoric correlation coefficients between measures of exposure and oblique promax rotation and the varimax prerotation method to make exposure factors more parsimonious [19]. For physiology variables, Spearman’s correlation coefficients were estimated and utilized a common exploratory factor analysis using the orthogonal parsimax rotation [20]. Factors were estimated with maximum likelihood methods [20,21]. As a sensitivity analysis, we implemented an alternative method for extracting factors: iterated principal factor analysis. We determined the number of factors to extract through parallel analysis, where each of the eigenvalues of the input correlation matrix was compared against an empirical distribution of eigenvalues. The empirical distribution of eigenvalues was obtained from 10,000 simulations of generated random correlation matrices. We retained all factors with corresponding eigenvalues that exceeded the one-sided critical value (α = 0.01) of the empirical eigenvalue distribution [20,22].

The eigenvalues and vectors were then used to compute the standardized (mean = 0, standard deviation = 1) latent constructs in the validation dataset, upon which multivariable-adjusted regression analysis that simultaneously adjusted for all of the exposure latent constructs could be applied to identify the structure of relationships between exposure latent constructs and latent constructs representing cardiometabolic health. These interrelationships were visualized using Cytoscape Version 3.7.2, with the following criteria dictating which associations were displayed: rotated factor pattern (standardized regression coefficients ≥ 0.5); uniqueness (display = all); inter-factor correlations (correlation coefficient ≥ 0.4); and multivariable-adjusted regression coefficients (significance under the Bonferroni criterion).

All analyses were conducted using SAS version 9.4, maintenance release #6.

## 3. Results

### 3.1. Participant Characteristics

All of the exposome variables that were included in the models are detailed in Table 1 and Supplementary Table S2. Of the 36,496 MVP participants analyzed, 86% were men, 85% were Caucasians and 11% were African-Americans (Table 1). The mean ± SD body mass index was 28 ± 5 kg/m^2^ (Supplementary Table S2). Markers of cardiometabolic health are presented in Table 2.

Two-thirds of participants (*n* = 24,411) were randomly assigned to the training dataset and the remaining one-third (*n* = 12,085) were randomly assigned to the validation dataset. The mean ± SD age of the participants in the training and validation datasets was identical (62.4 ± 13.4 years).

### 3.2. Latent Constructs Describing the Exposure Variables in the Training Dataset

**Figure 1:**
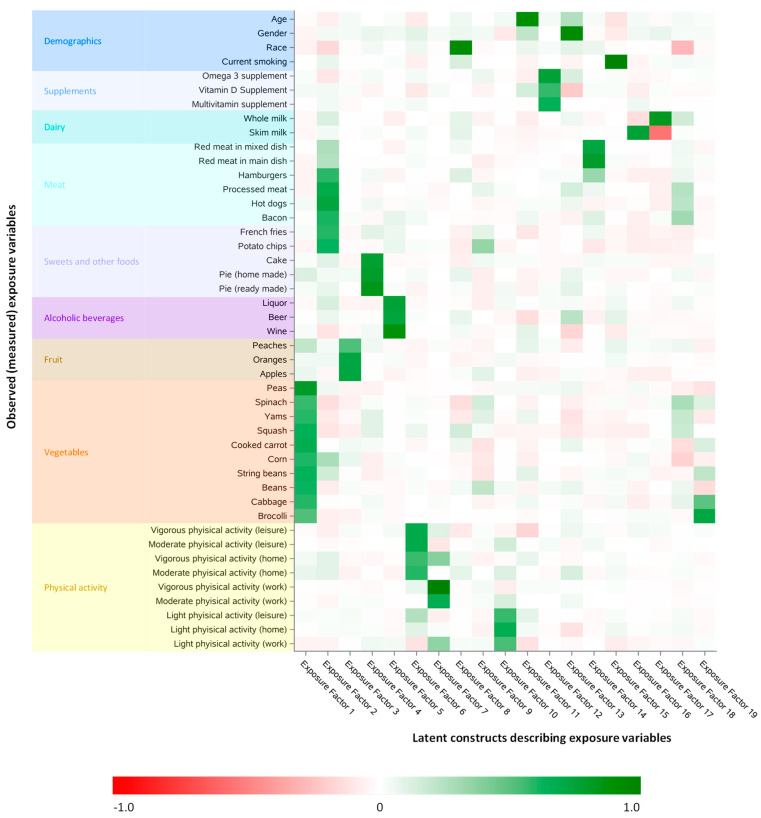
Rotated factor pattern based on tetrachoric and polychoric, common exploratory factor analysis of measured exposure variables in the training dataset; limited to observed (measured) variables that had a standardized regression coefficient ≥ 0.5 for at least one latent construct. These latent constructs (common factors) are those that best described the shared covariance of the observed (measured) exposures in the training, dataset.Number of participants: 24,411. Standardized regression coefficient.

Tetrachoric and polychoric, common exploratory factor analysis in the training dataset revealed 19 common factors that explained shared exposure observed (measured) variable covariance. The common factors could be broadly categorized according to the measured (observed) variables they represented (Figure 1 and Supplementary Figure S2). For example, the Common Exposure Factor E1 represented shared covariance in the intakes of many commonly consumed vegetables. Furthermore, Common Exposure Factor E17 had strong positive weighting for intake of whole milk but a strong negative weighting for intake of skim milk, representing the fact that, in this cohort, participants who frequently consumed whole milk were less likely to frequently consume skim milk.

Different types of physical activity were grouped together in three separate Common Exposure Factors. In particular, Common Exposure Factor E6 represented moderate and vigorous physical activity at home and during leisure time, Common Exposure Factor E7 represented moderate and vigorous physical activity at work, and Common Exposure Factor E10 represented light levels of physical activity at home, during leisure and at work.

### 3.3. Latent Constructs Describing the Physiological Variables in the Training Dataset

**Figure 2:**
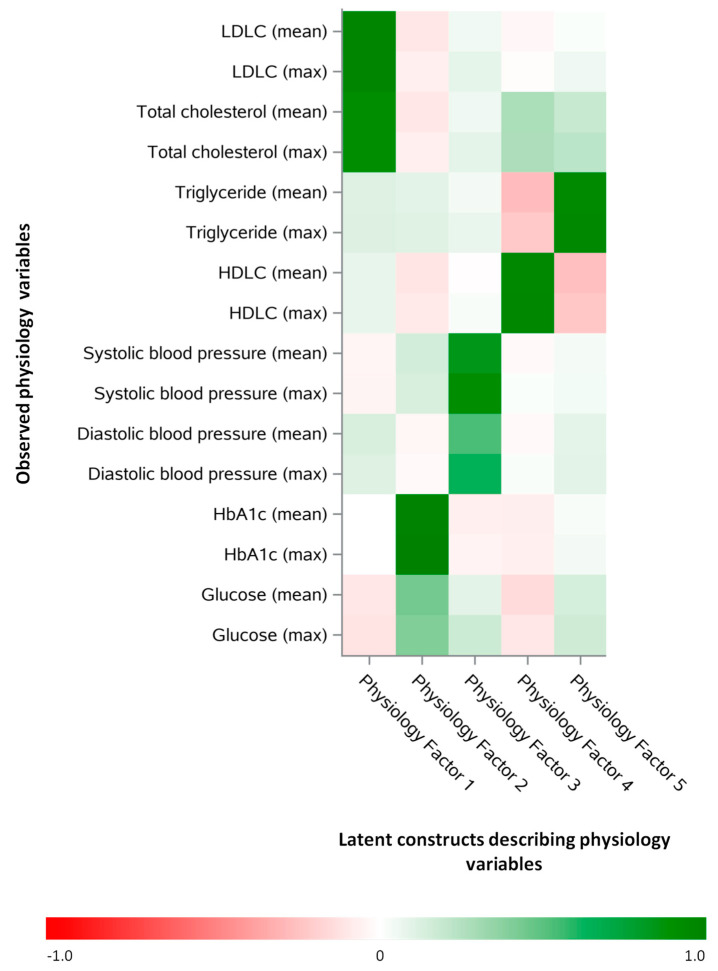
Rotated factor pattern based on common exploratory factor analysis of measured physiology variables in the training dataset. These latent constructs (common factors) are those that best described the shared covariance of the observed (measured) physiology variables in the training dataset.Number of participants: 24,411, “Mean” represents the mean measurement of all assessments. “Max” represents the maximum value of all the assessments. Abbreviations: LDLC: low-density lipoprotein cholesterol; HDLC: high-density lipoprotein cholesterol; HbA1c: glycated hemoglobin. Key:Standardized regression coefficient.

Common exploratory factor analysis in the training dataset revealed 5 common factors that explained shared physiology variable covariance. These broadly represented (i) total cholesterol and LDLC; (ii) glycemic control; (iii) blood pressure; (iv) HDLC; and (v) triglycerides (Figure 2). Common Physiology Factor P1 had positive loadings for all measures of total cholesterol and LDLC, and Common Physiology Factor P3 had high loadings for all of the measures of blood pressure. In fact, the final model applied similar loadings to mean and maximum values of the observed (measured) variables. As sensitivity analysis, we implemented an iterated principal factor analysis as the extraction method and observed similar factor loadings with the exception of mean and maximum glucose, which went from having a factor loading < 0.5 for Common Physiology Factor P2 in the primary analysis to having a factor loading > 0.5 (0.64 and 0.60, respectively) for Common Physiology Factor P2 in the sensitivity analysis.

### 3.4. Relationships between Human Exposures and Physiology in the Validation Dataset

**Figure 3:**
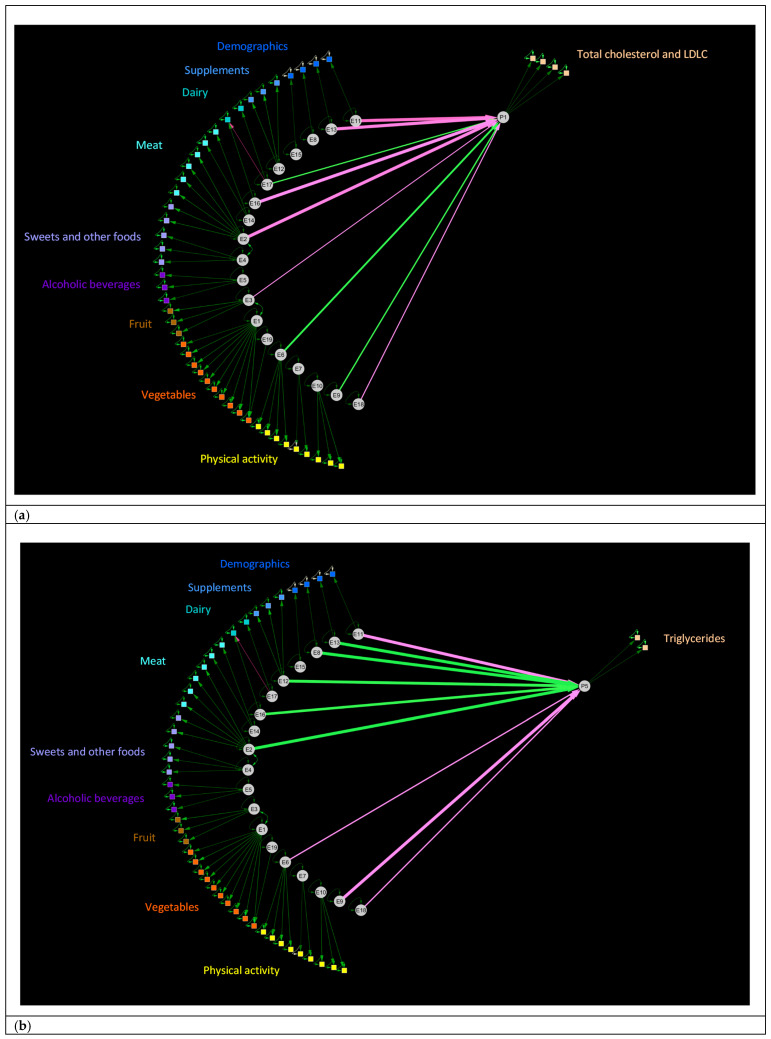

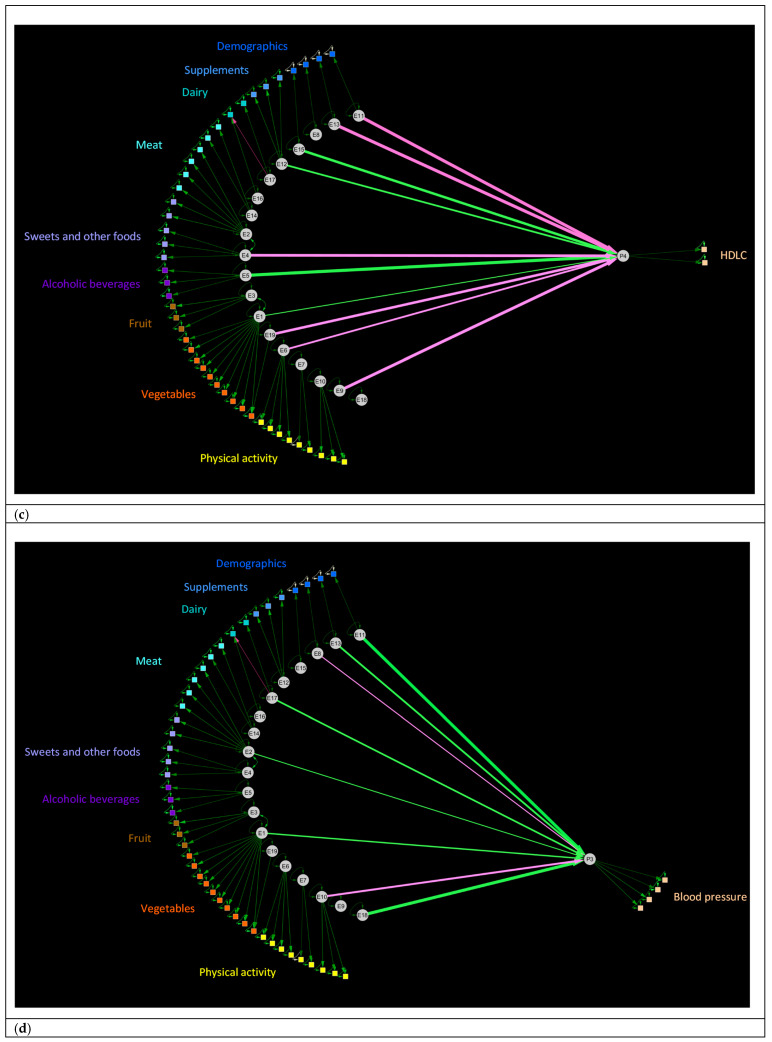

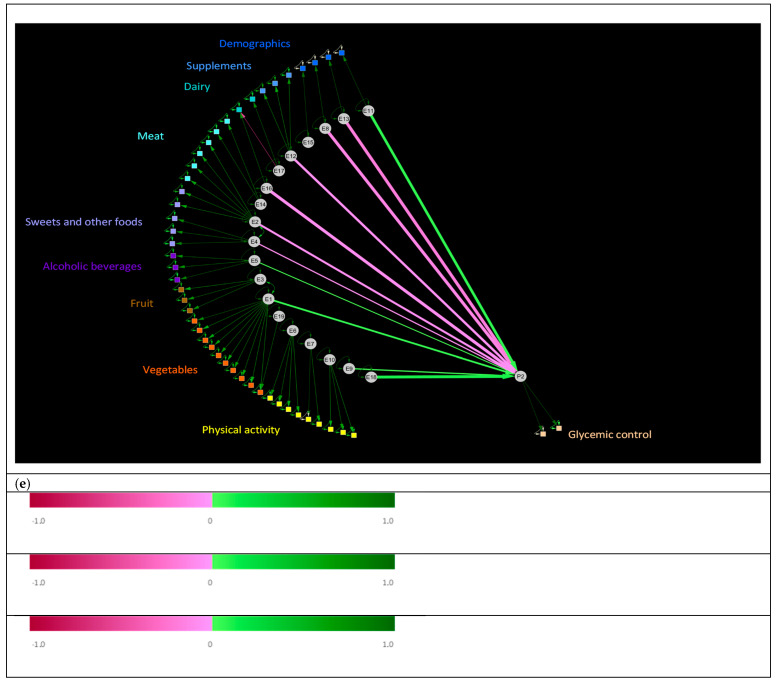
Common exploratory factor analysis (training dataset) and multiple regression analysis (validation dataset) outlining the interrelationships between human exposures and markers of cardiometabolic health. (**a**) Association of latent constructs representing exposure to various dietary and lifestyle common factors with the latent construct that explains the shared covariance in total cholesterol and low-density lipoprotein cholesterol (LDLC) concentrations. (**b**) Association of latent constructs representing exposure to various dietary and lifestyle common factors with the latent construct representing triglyceride concentrations. (**c**) Association of latent constructs representing exposure to various dietary and lifestyle common factors with the latent construct representing high-density lipoprotein cholesterol (HDLC) concentrations. (**d**) Association of latent constructs representing exposure to various dietary and lifestyle common factors with the latent construct representing blood pressure. (**e**) Association of latent constructs representing exposure to various dietary and lifestyle common factors with the latent construct representing glycemic control. Number of participants for the common exploratory factor analysis that was conducted in the training dataset: n = 24,411. Number of participants for the multivariable-adjusted regression analysis that was conducted in the validation dataset: n = 12,085. Observed (measured) exposure and physiology variables are presented in the order in which they appear in Figure 1 and Figure 2, respectively. Criteria for displaying measured (observed) variables: rotated factor pattern: Standardized regression coefficient ≥ 0.5. Criteria for displaying association lines: uniqueness (display all); inter-factor correlations (correlation coefficient ≥ 0.4); and multivariable, adjusted regression coefficients (*p* value significant using the Bonferroni threshold). For multivariable-adjusted regression coefficients, the line thickness represents the value of the -log10(*p* value), range: 2.11, 9.05.

Identification of the 19 Common Exposure Factors was done without knowledge of the physiological variables. Likewise, the creation of the 5 Common Physiology Factors was independent of the exposure variables. In Figure 3a–e, we present the complex patterns underlying the structure of relationships between Common Exposure Factors and Common Physiology Factors that remain after taking into account the non-independence of the assessed exposome. Some Common Exposure Factors had no association with the Common Physiology Factors, whereas others showed a strong association, both inversely and positively. Specifically, even though the Common Exposure Factor describing intake of processed meat and fried potato (E2) was associated with the Common Physiology Factors describing total cholesterol and LDLC (P1), triglycerides (P5), blood pressure (P3), and glycemic control (P2), the Common Exposure Factor representing red meat intake from main and mixed dishes (E14) was not associated with any of the physiological common factors. Similarly, although the Common Exposure Factor describing intake of moderate and vigorous physical activity at home and during leisure (E6) was associated with the Common Physiology Factors describing total cholesterol and LDLC (P1), triglycerides (P5), and HDLC (P4), the Common Exposure Factor representing moderate and vigorous physical activity at work (E7) was not associated with any of the physiological common factors.

When considering individual physiology factors, the fruit latent construct (Common Exposure Factor E3), but not the vegetable latent constructs (Common Exposure Factors E1 and E19) was inversely associated (estimate: −0.03, P: 0.0077) with the latent construct summarizing total cholesterol and LDLC (Common Physiology Factor P1) (Figure 3a). Conversely, the latent construct with a positive weighting for intake of whole milk but a strong negative weighting for intake of skim milk (Common Exposure Factor E17) had a positive association with Common Physiology Factor P1, as well as with Common Physiology Factor P3, the latent construct summarizing measures of blood pressure (Figure 3d). The latent construct describing light levels of physical activity (Common Exposure Factor E10) was inversely associated with the blood pressure latent construct.

## 4. Discussion

In this prospective cohort study of U.S. male and female Veterans, we reported an association between the exposome and markers of cardiometabolic health. Specifically, using factors identified in a training dataset, multiple multivariable-adjusted regression analyses revealed significant positive and inverse associations between exposure latent constructs and latent constructs describing observed (measured) physiology variables when applied to a separate validation dataset containing different participants. This provided us with critical insights and observations that represent steps forward in enhancing our understanding of how the exposome, as a holistic entity, shapes human physiology.

We employed common exploratory factor analysis to reveal the structure of interrelationships between individual exposures and physiology variables in a way that substantially advances our understanding of how observed (measured) exposome variables relate to the cardiometabolic risk profile. For example, a Common Exposure Factor was created to reflect the close relationship in study participants between high levels of moderate and vigorous physical activity at home and high levels of moderate and vigorous physical activity during leisure time. This relationship was not strongly correlated with levels of moderate and vigorous physical activity at work, which was represented by a different Common Exposure Factor. This suggests that the amount of moderate to vigorous physical activity participants perform at work did not covary with the amount of moderate to vigorous physical activity performed during leisure and at home [23]. By unveiling “hidden” relationships between the latent constructs and the observed (measured) variables they represent that matched our understanding of biology and variable representation, the utility of common exploratory factor analysis for both questionnaire-derived assessments of exposome and electronic medical record-derived assessments of cardiometabolic health was highlighted. However, it is unclear what the causal implications for these relationships are.

Very few studies have attempted to determine the influence of the exposome as a whole on cardiometabolic risk (as determined through electronic medical records). Modelling the system of relationships underlying the way in which the exposome, as a whole, shaped the cardiometabolic risk profile was therefore an important aim of our investigation. In addition to revealing the structure of the exposome and the structure of physiology variables, this study also revealed the structure of relationships between the exposome and cardiometabolic risk profile through multiple regression of latent constructs. An example of this was the creation of a latent construct in the training dataset that represented the reciprocal relationship between consumption of whole and skim milk. In other words, any associations of whole milk with cardiometabolic disease risk could not be separated from the effects of skim milk and should not be interpreted in isolation. When applied to separate validation datasets, this milk-based latent construct was positively associated with the latent constructs representing total cholesterol and LDLC as well as systolic and diastolic blood pressure. This observation is supported by randomized controlled trials directly comparing non-fermented whole milk to non-fermented skim milk that suggest adverse effects of whole milk, compared to skim milk, on total cholesterol and LDLC [24,25]. Further, skim but not whole milk has been shown to exhibit antihypertensive properties [26,27]. It is not yet clear whether dairy fat intake increases cardiovascular disease risk [28]. Despite this, results of our exposome analysis support the 2006 American Heart Association to Diet and Lifestyle Recommendations and the 2015–2020 Dietary Guidelines for Americans, which both encourage adults to select milk products that are either fat-free or low in fat rather than whole milk products [29,30]. The finding that individual components of the exposome are both numerically and biologically intertwined highlights the urgent need to implement analytic techniques that holistically examine the complex networks of interrelationships within and between observed (measured) variables. This was achieved through the representation of unmeasurable or unmeasured theoretical constructs as well as parameter-specific measurement error in order to draw robust generalizations regarding the complex interactions between the many exposome components and human physiology.

The use of latent factors to describe interrelationships between individual exposome and physiology components was able to shed light on hypothesized relationships. For example, the factor describing fruit consumption was inversely associated with the factor describing concentrations of total cholesterol and LDLC. This is supported by (i) our previous findings from the National Heart, Lung, and Blood Institute Family Heart Study, which found that consumption of fruit and vegetables was inversely related to LDLC in both men and women [31] and (ii) results from other cohort studies and randomized controlled trials [32,33]. Although the benefits of fruit consumption on cholesterol concentrations are not conclusive, with some studies showing no benefit of fruit consumption [34], the high fiber content of fruit has been attributed to its cholesterol-lowering capacity [35,36]. In this study, peaches, oranges, and apples contributed to the fruit factor. Apples have been shown to increase the clearance of plasma cholesterol by enhancing the fecal excretion of bile acids and cholesterol [37], and the peel of peaches has been shown to lower total cholesterol and LDLC in rats fed a high-sucrose diet. Further, the polyphenols in apples have been shown to have beneficial effects on cholesterol metabolism [38,39,40,41], as too have the pectins of apples and oranges [42]. A randomized controlled trial testing the effect of the combination of peaches, oranges, and apples on serum lipid profile is needed in order to ascertain causality of this observed association. It is important to note that there were cases where hypothesized relationships were not observed. For example, despite vegetables being a rich source of dietary fiber and higher consumption of vegetables being associated with lower risk of all-cause mortality and cardiovascular mortality [43], the vegetable consumption factors in this study were not significantly associated with the factor describing total cholesterol and LDLC. The absence of confirmatory findings regarding vegetables in this study may be explained by the absence of data on intake of nutrients, such as fiber, which can summarize contributions from many different foods that are biologically important. Another reason may be that the measurement error may be lower in fruits as opposed to vegetables. However, another interpretation may be that, after controlling for other exposome components, vegetables are not associated with total cholesterol and LDLC concentrations in this cohort. Further studies using longitudinal data are needed to confirm these findings.

Although the methods implemented were well-tested and effective, it is important to note that diet was self-reported, health outcomes were captured through electronic medical records, there was a lack of data on medication adherence, and there were a limited number of women and non-whites in this U.S. Veteran cohort. Additionally, causality of observed relationships could not be established due to the observational nature of the study. Nevertheless, it is important to note that the exposome was measured at least one year prior to any of the physiologic variables being assessed, which, although not ruling it out, does reduce the likelihood and impact of reverse causation. An additional factor to consider when interpreting the results is the possibility of false-positive findings, which was reduced through implementation of the conservative Bonferroni correction [44]. Furthermore, although residual or unmeasured confounders cannot be ruled out, the common exploratory methods implemented in this study do aim to represent unmeasured and/or immeasurable variables through the creation of latent constructs. This analytic approach also enabled us to model the measurement error inherent when using self-reported exposome assessments as well as collations from electronic medical records, even when there are some exposome variables, such as environmental variables, that are not measured directly. By conducting the analysis in both a training and a validation dataset, we were able to demonstrate the utility of our analytic strategy for use in the increasingly prevalent type of cohort that has extensive questionnaire-based assessments of the exposome as well as markers of human physiology derived from electronic medical records. However, further replication in separate datasets is warranted.

It is becoming increasingly recognized that studies of the complete exposome are more biologically representative than fragmented models based on subsets of factors. There is no more clear example of this than the position of the Academy of Nutrition and Dietetics, which plainly states that the “total diet or overall pattern of food eaten is the most important focus of healthy eating” [45]. In recognition of this, as opposed to focusing on individual nutrient recommendations, the Dietary Guidelines for Americans highlight key elements of healthy eating patterns [30]. Some patterns, such as the Mediterranean Dietary Pattern, are based on a priori knowledge of how individual dietary components influence human health, and are often represented by a pattern score that reflects relative adherence to the dietary pattern, as is the case for the Healthy Eating Index [46,47]. Although hypothesis-driven, there is no general consensus in the scientific or clinical community as to what is the ideal dietary pattern for optimal health [48]. Importantly, these dietary patterns typically reflect only a select group of dietary components, and not necessarily the diet as a whole [46,47]. Given that the aim of the present paper was to identify the importance of the entire exposome in influencing cardiometabolic health, we chose to adopt a data-driven approach which allowed us to identify existing patterns in the population, and how individual foods, lifestyle factors, and demographic features related to each other at a population level. In this study, the measured exposome variables were represented by 19 common factors that were created independently from the physiological variables. We observed heterogeneous patterns of association of exposome constructs with each of the physiological constructs, which represented measured biomarkers that are important indicators of cardiometabolic health. As such, even though the exposome overall contributed to cardiometabolic health, each facet of the exposome had differential implications for different aspects of cardiometabolic health. It is evident that the overall complex exposome that individuals are exposed to needs to be studied more in order to more fully understand what is contributing to cardiometabolic risk profiles in communities of free-living individuals. To be more comprehensive, a logical extension to this current work would be to incorporate more environmental exposome variables, such as air pollution and access to green space, into the current models.

In conclusion, we observed a complex pattern of associations between the exposome and markers of cardiometabolic health. Given that we are increasingly recognizing the potential of the exposome as a whole to have far-reaching health implications beyond the effects associated with the sum of its parts, it is more important than ever that we make available analytic tools and approaches that are capable of dealing with this directly. It should be noted that the analytic strategy implemented in this paper could be applied to address a range of research questions that utilize data from questionnaire and electronic health record data, thus bringing us one step closer to understanding how the exposome, as a whole, impacts human health.

## Figures and Tables

**Table 1:** Key baseline characteristics of all Million Veteran Program participants included in this study.

	Value
**DEMOGRAPHICS**	
Age (years)	62.40 ± 13.41
Gender (% males)	86
Caucasian (%)	85
Current smoking (number of cigarettes smoked/day)	0.26 ± 0.77
**SUPPLEMENT USE**	
Omega-3 supplement use (%)	23
Vitamin D supplement use (%)	36
Multivitamin supplement use (%)	54
**DIETARY INTAKE**	
**Dairy**	
Whole milk (serves/day)	0.17 ± 0.55
Skim milk (serves/day)	0.51 ± 0.87
**Meat**	
Red meat in main dish (serves/day)	0.20 ± 0.28
Red meat in mixed dish (serves/day)	0.19 ± 0.26
Hamburgers (serves/day)	0.16 ± 0.24
Processed meat (serves/day)	0.16 ± 0.28
Hot dogs (serves/day)	0.08 ± 0.19
Bacon (serves/day)	0.15 ± 0.29
**Sweets and other foods**	
French fries (serves/day)	0.12 ± 0.23
Potato chips (serves/day)	0.18 ± 0.32
Cake (serves/day)	0.06 ± 0.15
Home-made pie (serves/day)	0.05 ± 0.13
Ready-made pie (serves/day)	0.05 ± 0.14
**Alcoholic beverages**	
Liquor (serves/day)	0.15 ± 0.52
Beer (serves/day)	0.31 ± 0.84
Wine (serves/day)	0.17 ± 0.49
**Fruit**	
Peaches (serves/day)	0.13 ± 0.32
Oranges (serves/day)	0.19 ± 0.37
Apples (serves/day)	0.27 ± 0.43
**Vegetables**	
Peas (serves/day)	0.14 ± 0.25
Spinach (serves/day)	0.15 ± 0.32
Yams (serves/day)	0.09 ± 0.23
Squash (serves/day)	0.07 ± 0.21
Cooked carrot (serves/day)	0.13 ± 0.25
Corn (serves/day)	0.15 ± 0.25
String beans (serves/day)	0.17 ± 0.26
Beans (serves/day)	0.18 ± 0.32
Cabbage (serves/day)	0.14 ± 0.28
Broccoli (serves/day)	0.19 ± 0.31
**PHYSICAL ACTIVITY**	
Vigorous physical activity during leisure time (hours/day)	0.85 ± 1.68
Moderate physical activity during leisure time (hours/day)	1.20 ± 1.92
Vigorous physical activity at home (hours/day)	0.85 ± 1.51
Moderate physical activity at home (hours/day)	1.14 ± 1.75
Vigorous physical activity at work (hours/day)	0.9 ± 1.87
Moderate physical activity at work (hours/day)	1.77 ± 2.53
Light physical activity during leisure time (hours/day)	2.37 ± 2.65
Light physical activity at home (hours/day)	2.93 ± 2.71
Light physical activity at work (hours/day)	2.96 ± 3.19

Number of participants: 36,496. Results are mean ± standard deviation or %, where appropriate.

**Table 2:** Physiological markers of cardiometabolic health in all included Million Veteran Program participants.

	Mean ± SD
**Total cholesterol**	
Mean of measurements (mg/dL)	177.40 ± 33.96
Maximum measurement (mg/dL)	187.28 ± 36.98
**Low-density lipoprotein cholesterol**	
Mean of measurements (mg/dL)	104.57 ± 29.36
Maximum measurement (mg/dL)	113.15 ± 31.90
**Triglycerides**	
Mean of measurements (mg/dL)	123.48 ± 63.96
Maximum measurement (mg/dL)	145.81 ± 80.31
**High-density lipoprotein cholesterol**	
Mean of measurements (mg/dL)	49.79 ± 13.94
Maximum measurement (mg/dL)	53.02 ± 15.10
**Systolic blood pressure**	
Mean of measurements (mmHg)	130.27 ± 12.43
Maximum measurement (mmHg)	145.71 ± 17.93
**Diastolic blood pressure**	
Mean of measurements (mmHg)	76.72 ± 7.67
Maximum measurement (mmHg)	85.74 ± 10.24
**HbA1c**	
Mean of measurements (DCCT %)	5.64 ± 0.58
Maximum measurement (DCCT %)	5.74 ± 0.66
**Glucose**	
Mean of measurements (mg/dL)	101.92 ± 17.70
Maximum measurement (mg/dL)	113.45 ± 26.93

Number of participants: 36,496. Mean of measurements reflects mean value for all measurements, whereas maximum measurement reflects the maximum value for all measurements.

## Data Availability

Data described in the article, code book, and analytic code will not be made available to other researchers for purposes of reproducing the results or replicating the procedure, in order to comply with current VA privacy regulations pursuant to the US Department of Veterans Administration policies on compliance with the confidentiality of US veterans’ data.

## Supplementary Materials

The following are available online at https://www.mdpi.com/article/10.3390/nu13041364/s1, Table S1: Number of participants excluded based on use of antilipemic, antihypertensive and/or hypoglycemic medications during either the exposure-assessment or outcome-assessment periods, Table S2: Additional baseline characteristics of all Million Veterans Program participants included in this study, Figure S1: Schematic overview of the training and validation datasets, Figure S2: Rotated factor pattern based on tetrachoric and polychoric, common exploratory factor analysis of measured exposure variables in the training dataset; limited to observed (measured) variables that did not have a standardized regression coefficient ≥ 0.5 for at least one latent construct.

## Appendix A

*MVP Executive Committee*

- Co-Chair: J. Michael Gaziano, M.D., M.P.H.
  - VA Boston Healthcare System, 150 S. Huntington Avenue, Boston, MA 02130, USA
- Co-Chair: Sumitra Muralidhar, Ph.D.
  - US Department of Veterans Affairs, 810 Vermont Avenue NW, Washington, DC 20420, USA
- Rachel Ramoni, D.M.D., Sc.D., Chief VA Research and Development Officer
  - US Department of Veterans Affairs, 810 Vermont Avenue NW, Washington, DC 20420, USA
- Jean Beckham, Ph.D.
  - Durham VA Medical Center, 508 Fulton Street, Durham, NC 27705, USA
- Kyong-Mi Chang, M.D.
  - Philadelphia VA Medical Center, 3900 Woodland Avenue, Philadelphia, PA 19104, USA
- Christopher J. O’Donnell, M.D., M.P.H.
  - VA Boston Healthcare System, 150 S. Huntington Avenue, Boston, MA 02130, USA
- Philip S. Tsao, Ph.D.
  - VA Palo Alto Health Care System, 3801 Miranda Avenue, Palo Alto, CA 94304, USA
- James Breeling, M.D., Ex-Officio
  - US Department of Veterans Affairs, 810 Vermont Avenue NW, Washington, DC 20420, USA
- Grant Huang, Ph.D., Ex-Officio
  - US Department of Veterans Affairs, 810 Vermont Avenue NW, Washington, DC 20420, USA
- Juan P. Casas, M.D., Ph.D., Ex-Officio
  - VA Boston Healthcare System, 150 S. Huntington Avenue, Boston, MA 02130, USA

*MVP Program Office*

- Sumitra Muralidhar, Ph.D.
  ∘ US Department of Veterans Affairs, 810 Vermont Avenue NW, Washington, DC 20420, USA
- Jennifer Moser, Ph.D.
  ∘ US Department of Veterans Affairs, 810 Vermont Avenue NW, Washington, DC 20420, USA

*MVP Recruitment/Enrollment*

- Recruitment/Enrollment Director/Deputy Director, Boston—Stacey B. Whitbourne, Ph.D.; Jessica V. Brewer, M.P.H.
  - VA Boston Healthcare System, 150 S. Huntington Avenue, Boston, MA 02130, USA
- MVP Coordinating Centers
  - Clinical Epidemiology Research Center (CERC), West Haven—Mihaela Aslan, Ph.D.
  - West Haven VA Medical Center, 950 Campbell Avenue, West Haven, CT 06516, USA
  - Cooperative Studies Program Clinical Research Pharmacy Coordinating Center, Albuquerque—Todd Connor, Pharm.D.; Dean P. Argyres, B.S., M.S.
  - New Mexico VA Health Care System, 1501 San Pedro Drive SE, Albuquerque, NM 87108, USA
  - Genomics Coordinating Center, Palo Alto—Philip S. Tsao, Ph.D.
  - VA Palo Alto Health Care System, 3801 Miranda Avenue, Palo Alto, CA 94304, USA
  - MVP Boston Coordinating Center, Boston—J. Michael Gaziano, M.D., M.P.H.
  - VA Boston Healthcare System, 150 S. Huntington Avenue, Boston, MA 02130, USA
  - MVP Information Center, Canandaigua—Brady Stephens, M.S.
  - Canandaigua VA Medical Center, 400 Fort Hill Avenue, Canandaigua, NY 14424, USA
- VA Central Biorepository, Boston—Mary T. Brophy M.D., M.P.H.; Donald E. Humphries, Ph.D.; Luis E. Selva, Ph.D.
  - VA Boston Healthcare System, 150 S. Huntington Avenue, Boston, MA 02130, USA
- MVP Informatics, Boston—Nhan Do, M.D.; Shahpoor (Alex) Shayan, M.S.
  - VA Boston Healthcare System, 150 S. Huntington Avenue, Boston, MA 02130, USA
- MVP Data Operations/Analytics, Boston—Kelly Cho, M.P.H., Ph.D.
  - VA Boston Healthcare System, 150 S. Huntington Avenue, Boston, MA 02130, USA
- Director of Regulatory Affairs—Lori Churby, B.S.
  - VA Palo Alto Health Care System, 3801 Miranda Avenue, Palo Alto, CA 94304, USA

*MVP Science*

- Science Operations—Christopher J. O’Donnell, M.D., M.P.H.
- VA Boston Healthcare System, 150 S. Huntington Avenue, Boston, MA 02130, USA
- Genomics Core—Christopher J. O’Donnell, M.D., M.P.H.; Saiju Pyarajan Ph.D.
- VA Boston Healthcare System, 150 S. Huntington Avenue, Boston, MA 02130, USA
- Philip S. Tsao, Ph.D.
- VA Palo Alto Health Care System, 3801 Miranda Avenue, Palo Alto, CA 94304, USA
- Data Core—Kelly Cho, M.P.H, Ph.D.
- VA Boston Healthcare System, 150 S. Huntington Avenue, Boston, MA 02130, USA
- VA Informatics and Computing Infrastructure (VINCI)—Scott L. DuVall, Ph.D.
- VA Salt Lake City Health Care System, 500 Foothill Drive, Salt Lake City, UT 84148, USA
- Data and Computational Sciences—Saiju Pyarajan, Ph.D.
- VA Boston Healthcare System, 150 S. Huntington Avenue, Boston, MA 02130, USA
- Statistical Genetics—Elizabeth Hauser, Ph.D.
- Durham VA Medical Center, 508 Fulton Street, Durham, NC 27705, USA
- Yan Sun, Ph.D.
- Atlanta VA Medical Center, 1670 Clairmont Road, Decatur, GA 30033, USA
- Hongyu Zhao, Ph.D.
- West Haven VA Medical Center, 950 Campbell Avenue, West Haven, CT 06516, USA

*Current MVP Local Site Investigators*

- Atlanta VA Medical Center (Peter Wilson, M.D.)
  ∘ 1670 Clairmont Road, Decatur, GA 30033, USA
- Bay Pines VA Healthcare System (Rachel McArdle, Ph.D.)
  ∘ 10,000 Bay Pines Blvd Bay Pines, FL 33744, USA
- Birmingham VA Medical Center (Louis Dellitalia, M.D.)
  ∘ 700 S. 19th Street, Birmingham, AL 35233, USA
- Central Western Massachusetts Healthcare System (Kristin Mattocks, Ph.D., M.P.H.)
  ∘ 421 North Main Street, Leeds, MA 01053, USA
- Cincinnati VA Medical Center (John Harley, M.D., Ph.D.)
  ∘ 3200 Vine Street, Cincinnati, OH 45220, USA
- Clement J. Zablocki VA Medical Center (Jeffrey Whittle, M.D., M.P.H.)
  ∘ 5000 West National Avenue, Milwaukee, WI 53295, USA
- VA Northeast Ohio Healthcare System (Frank Jacono, M.D.)
  ∘ 10701 East Boulevard, Cleveland, OH 44106, USA
- Durham VA Medical Center (Jean Beckham, Ph.D.)
  ∘ 508 Fulton Street, Durham, NC 27705, USA
- Edith Nourse Rogers Memorial Veterans Hospital (John Wells., Ph.D.)
  ∘ 200 Springs Road, Bedford, MA 01730, USA
- Edward Hines, Jr. VA Medical Center (Salvador Gutierrez, M.D.)
  ∘ 5000 South 5th Avenue, Hines, IL 60141, USA
- Veterans Health Care System of the Ozarks (Gretchen Gibson, D.D.S., M.P.H.)
  ∘ 1100 North College Avenue, Fayetteville, AR 72703, USA
- Fargo VA Health Care System (Kimberly Hammer, Ph.D.)
  ∘ 2101 N. Elm, Fargo, ND 58102, USA
- VA Health Care Upstate New York (Laurence Kaminsky, Ph.D.)
  ∘ 113 Holland Avenue, Albany, NY 12208, USA
- New Mexico VA Health Care System (Gerardo Villareal, M.D.)
  ∘ 1501 San Pedro Drive, S.E. Albuquerque, NM 87108, USA
- VA Boston Healthcare System (Scott Kinlay, M.B.B.S., Ph.D.)
  ∘ 150 S. Huntington Avenue, Boston, MA 02130, USA
- VA Western New York Healthcare System (Junzhe Xu, M.D.)
  ∘ 3495 Bailey Avenue, Buffalo, NY 14215-1199, USA
- Ralph H. Johnson VA Medical Center (Mark Hamner, M.D.)
  ∘ 109 Bee Street, Mental Health Research, Charleston, SC 29401
- Columbia VA Health Care System (Roy Mathew, M.D.)
  ∘ 6439 Garners Ferry Road, Columbia, SC 29209, USA
- VA North Texas Health Care System (Sujata Bhushan, M.D.)
  ∘ 4500 S. Lancaster Road, Dallas, TX 75216, USA
- Hampton VA Medical Center (Pran Iruvanti, D.O., Ph.D.)
  ∘ 100 Emancipation Drive, Hampton, VA 23667, USA
- Richmond VA Medical Center (Michael Godschalk, M.D.)
  ∘ 1201 Broad Rock Blvd., Richmond, VA 23249, USA
- Iowa City VA Health Care System (Zuhair Ballas, M.D.)
  ∘ 601 Highway 6 West, Iowa City, IA 52246-2208
- Eastern Oklahoma VA Health Care System (Douglas Ivins, M.D.)
  ∘ 1011 Honor Heights Drive, Muskogee, OK 74401, USA
- James A. Haley Veterans’ Hospital (Stephen Mastorides, M.D.)
  ∘ 13000 Bruce B. Downs Blvd, Tampa, FL 33612, USA
- James H. Quillen VA Medical Center (Jonathan Moorman, M.D., Ph.D.)
  ∘ Corner of Lamont & Veterans Way, Mountain Home, TN 37684, USA
- John D. Dingell VA Medical Center (Saib Gappy, M.D.)
  ∘ 4646 John R Street, Detroit, MI 48201, USA
- Louisville VA Medical Center (Jon Klein, M.D., Ph.D.)
  ∘ 800 Zorn Avenue, Louisville, KY 40206, USA
- Manchester VA Medical Center (Nora Ratcliffe, M.D.)
  ∘ 718 Smyth Road, Manchester, NH 03104, USA
- Miami VA Health Care System (Hermes Florez, M.D., Ph.D.)
  ∘ 1201 NW 16th Street, 11 GRC, Miami, FL 33125, USA
- Michael E. DeBakey VA Medical Center (Olaoluwa Okusaga, M.D.)
  ∘ 2002 Holcombe Blvd, Houston, TX 77030, USA
- Minneapolis VA Health Care System (Maureen Murdoch, M.D., M.P.H.)
  ∘ One Veterans Drive, Minneapolis, MN 55417, USA
- N. FL/S. GA Veterans Health System (Peruvemba Sriram, M.D.)
  ∘ 1601 SW Archer Road, Gainesville, FL 32608, USA
- Northport VA Medical Center (Shing Shing Yeh, Ph.D., M.D.)
  ∘ 79 Middleville Road, Northport, NY 11768, USA
- Overton Brooks VA Medical Center (Neeraj Tandon, M.D.)
  ∘ 510 East Stoner Ave, Shreveport, LA 71101, USA
- Philadelphia VA Medical Center (Darshana Jhala, M.D.)
  ∘ 3900 Woodland Avenue, Philadelphia, PA 19104, USA
- Phoenix VA Health Care System (Samuel Aguayo, M.D.)
  ∘ 650 E. Indian School Road, Phoenix, AZ 85012, USA
- Portland VA Medical Center (David Cohen, M.D.)
  ∘ 3710 SW U.S. Veterans Hospital Road, Portland, OR 97239
- Providence VA Medical Center (Satish Sharma, M.D.)
  ∘ 830 Chalkstone Avenue, Providence, RI 02908, USA
- Richard Roudebush VA Medical Center (Suthat Liangpunsakul, M.D., M.P.H.)
  ∘ 1481 West 10th Street, Indianapolis, IN 46202, USA
- Salem VA Medical Center (Kris Ann Oursler, M.D.)
  ∘ 1970 Roanoke Blvd, Salem, VA 24153, USA
- San Francisco VA Health Care System (Mary Whooley, M.D.)
  ∘ 4150 Clement Street, San Francisco, CA 94121, USA
- South Texas Veterans Health Care System (Sunil Ahuja, M.D.)
  ∘ 7400 Merton Minter Boulevard, San Antonio, TX 78229, USA
- Southeast Louisiana Veterans Health Care System (Joseph Constans, Ph.D.)
  ∘ 2400 Canal Street, New Orleans, LA 70119, USA
- Southern Arizona VA Health Care System (Paul Meyer, M.D., Ph.D.)
  ∘ 3601 S 6th Avenue, Tucson, AZ 85723, USA
- Sioux Falls VA Health Care System (Jennifer Greco, M.D.)
  ∘ 2501 W 22nd Street, Sioux Falls, SD 57105, USA
- St. Louis VA Health Care System (Michael Rauchman, M.D.)
  ∘ 915 North Grand Blvd, St. Louis, MO 63106, USA
- Syracuse VA Medical Center (Richard Servatius, Ph.D.)
  ∘ 800 Irving Avenue, Syracuse, NY 13210, USA
- VA Eastern Kansas Health Care System (Melinda Gaddy, Ph.D.)
  ∘ 4101 S 4th Street Trafficway, Leavenworth, KS 66048, USA
- VA Greater Los Angeles Health Care System (Agnes Wallbom, M.D., M.S.)
  ∘ 11301 Wilshire Blvd, Los Angeles, CA 90073, USA
- VA Long Beach Healthcare System (Timothy Morgan, M.D.)
  ∘ 5901 East 7th Street Long Beach, CA 90822, USA
- VA Maine Healthcare System (Todd Stapley, D.O.)
  ∘ 1 VA Center, Augusta, ME 04330, USA
- VA New York Harbor Healthcare System (Scott Sherman, M.D., M.P.H.)
  ∘ 423 East 23rd Street, New York, NY 10010, USA
- VA Pacific Islands Health Care System (George Ross, M.D.)
  ∘ 459 Patterson Rd, Honolulu, HI 96819, USA
- VA Palo Alto Health Care System (Philip Tsao, Ph.D.)
  ∘ 3801 Miranda Avenue, Palo Alto, CA 94304-1290, USA
- VA Pittsburgh Health Care System (Patrick Strollo, Jr., M.D.)
  ∘ University Drive, Pittsburgh, PA 15240, USA
- VA Puget Sound Health Care System (Edward Boyko, M.D.)
  ∘ 1660 S. Columbian Way, Seattle, WA 98108-1597, USA
- VA Salt Lake City Health Care System (Laurence Meyer, M.D., Ph.D.)
  ∘ 500 Foothill Drive, Salt Lake City, UT 84148, USA
- VA San Diego Healthcare System (Samir Gupta, M.D., M.S.C.S.)
  ∘ 3350 La Jolla Village Drive, San Diego, CA 92161, USA
- VA Sierra Nevada Health Care System (Mostaqul Huq, Pharm.D., Ph.D.)
  ∘ 975 Kirman Avenue, Reno, NV 89502, USA
- VA Southern Nevada Healthcare System (Joseph Fayad, M.D.)
  ∘ 6900 North Pecos Road, North Las Vegas, NV 89086, USA
- VA Tennessee Valley Healthcare System (Adriana Hung, M.D., M.P.H.)
  ∘ 1310 24th Avenue, South Nashville, TN 37212, USA
- Washington DC VA Medical Center (Jack Lichy, M.D., Ph.D.)
  ∘ 50 Irving St, Washington, D. C. 20422, USA
- W.G. (Bill) Hefner VA Medical Center (Robin Hurley, M.D.)
  ∘ 1601 Brenner Ave, Salisbury, NC 28144, USA
- White River Junction VA Medical Center (Brooks Robey, M.D.)
  ∘ 163 Veterans Drive, White River Junction, VT 05009, USA
- William S. Middleton Memorial Veterans Hospital (Robert Striker, M.D., Ph.D.)
  ∘ 2500 Overlook Terrace, Madison, WI 53705, USA
