# Supplementary material for: The Structure of Relationships between the Human Exposome and Cardiometabolic Health: The Million Veteran Program
Source: Nutrients. 2021 Apr 19;13(4):1364. doi: 10.3390/nu13041364 (PMC8073795; doi:10.3390/nu13041364)

The structure of relationships between the human exposome and cardiometabolic health: The Million Veteran Program

SUPPLEMENTARY INFORMATION

Table S1: Number of participants excluded based on use of antilipemic, antihypertensive and/or hypoglycemic medications during either the exposure-assessment or outcome-assessment periods

	**Number excluded**
**Use of antilipemic, antihypertensive and hypoglycemic medications**	14,943
**Use of antilipemic and antihypertensive medications**	12,286
**Use of antilipemic and hypoglycemic medications**	53,776
**Use of antihypertensive and hypoglycemic medications**	1,471
**Use of antilipemic medications only**	85,233
**Use of antihypertensive medications only**	5,940
**Use of hypoglycemic medications only**	8,714

Table S2: Additional baseline characteristics of all Million Veterans Program participants included in this study

	Value
**DEMOGRAPHICS**	
Previous smoking *(number of cigarettes smoked/day)*	1.37 ± 1.52
Hispanic or Latino *(%)*	6
Body mass index *(kg/m²)*	27.91 ± 5.37
**DIETARY INTAKE**	
**Dairy**	
Yogurt *(serves/day)*	0.23 ± 0.46
Ice cream *(serves/day)*	0.20 ± 0.38
Cottage Cheese *(serves/day)*	0.08 ± 0.24
Other cheese *(serves/day)*	0.46 ± 0.58
Butter *(serves/day)*	0.39 ± 0.68
**Meat**	
Eggs *(serves/day)*	0.43 ± 0.57
Chicken with skin on *(serves/day)*	0.25 ± 0.40
Chicken without skin *(serves/day)*	0.27 ± 0.36
Liver *(serves/day)*	0.01 ± 0.11
Fish *(serves/day)*	0.17 ± 0.28
**Sweets and other foods**	
Nuts *(serves/day)*	0.30 ± 0.51
Peanut butter *(serves/day)*	0.25 ± 0.43
Chocolate *(serves/day)*	0.28 ± 0.50
Candy *(serves/day)*	0.14 ± 0.39
Cookies *(serves/day)*	0.22 ± 0.48
Margarine *(serves/day)*	0.32 ± 0.61
Dressing *(serves/day)*	0.21 ± 0.37

**Grains**	
Cold cereal *(serves/day)*	0.33 ± 0.47
White bread *(serves/day)*	0.27 ± 0.55
Dark bread *(serves/day)*	0.39 ± 0.60
Rice *(serves/day)*	0.27 ± 0.36
**Non-alcoholic beverages**	
Coffee *(serves/day)*	1.30 ± 1.46
Tea *(serves/day)*	0.39 ± 0.85
Diet carbonated beverages *(serves/day)*	0.27 ± 0.72
Sugar carbonated beverages *(serves/day)*	0.22 ± 0.60
Fruit drinks *(serves/day)*	0.17 ± 0.48
Orange or grapefruit juice *(serves/day)*	0.27 ± 0.5
**Vegetables**	
Tomato *(serves/day)*	0.31 ± 0.44
Raw carrot *(serves/day)*	0.20 ± 0.37
Baked potato *(serves/day)*	0.22 ± 0.29
**Fruit**	
Bananas *(serves/day)*	0.41 ± 0.53
Other fruit *(serves/day)*	0.36 ± 0.56
**PHYSICAL INACTIVITY**	
Playing video games *(hours/week)*	1.55 ± 6.81
Using a computer *(hours/week)*	16.19 ± 21.27
Watching TV *(hours/week)*	21.36 ± 21.78

Number of participants: 36,496
Results are mean ± standard deviation or %, where appropriate

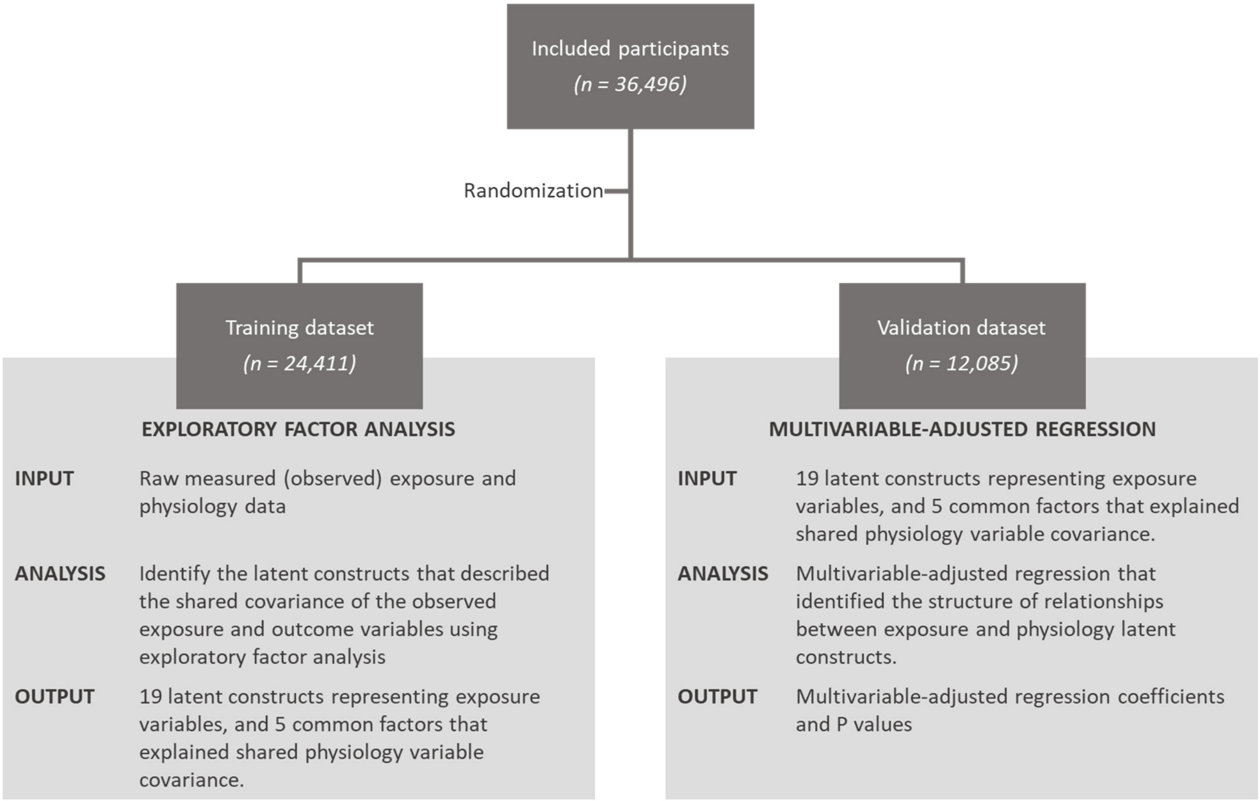

Figure S1: Schematic overview of the training and validation datasets.

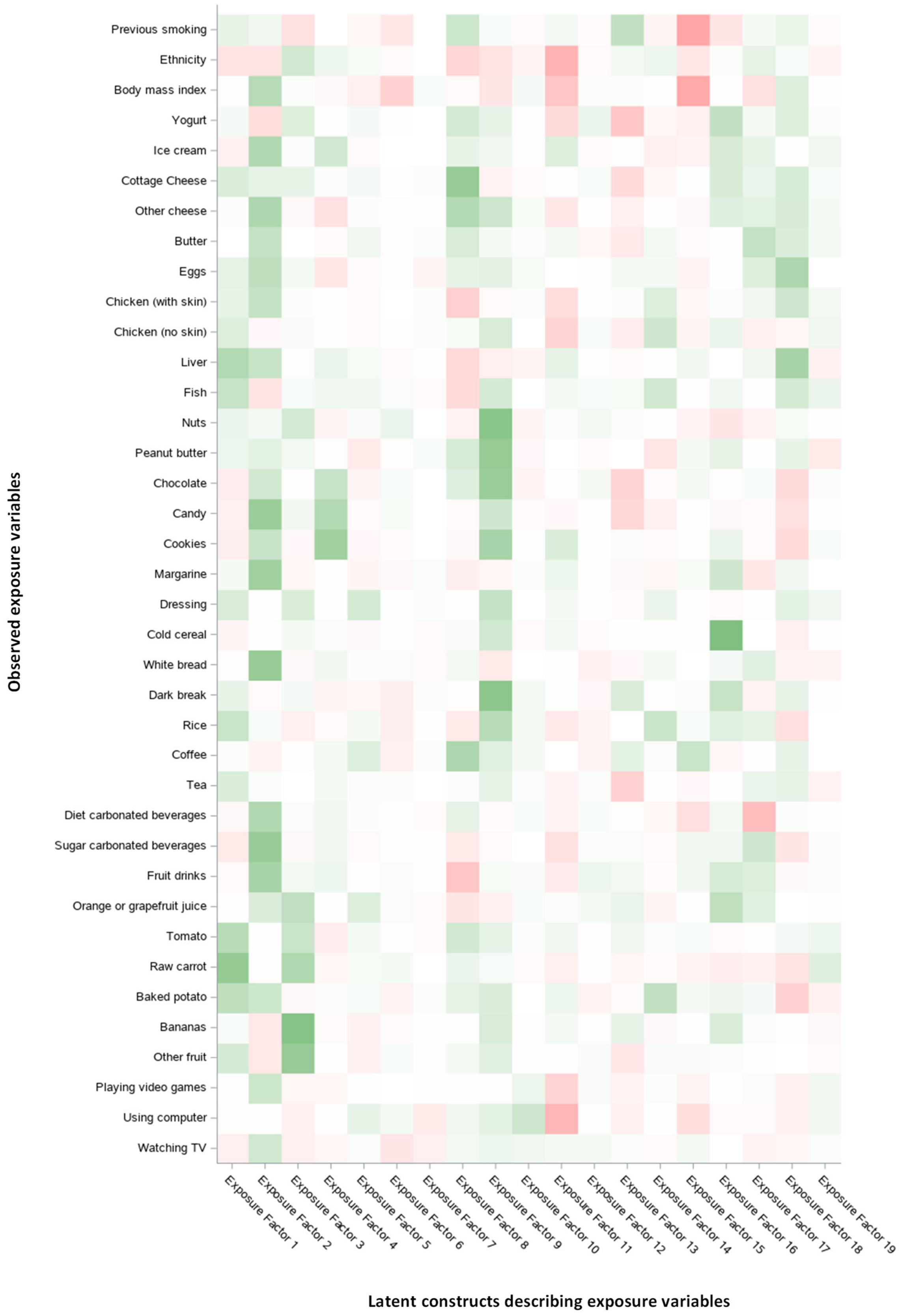
Observed exposure variables
Previous smoking
Ethnicity
Body mass index
Yogurt
Ice cream
Cottage Cheese
Other cheese
Butter
Eggs
Chicken (with skin)
Chicken (no skin)
Liver
Fish
Nuts
Peanut butter
Chocolate
Candy
Cookies
Margarine
Dressing
Cold cereal
White bread
Dark break
Rice
Coffee
Tea
Diet carbonated beverages
Sugar carbonated beverages
Fruit drinks
Orange or grapefruit juice
Tomato
Raw carrot
Baked potato
Bananas
Other fruit
Playing video games
Using computer
Watching TV
Exposure Factor 1
Exposure Factor 2
Exposure Factor 3
Exposure Factor 4
Exposure Factor 5
Exposure Factor 6
Exposure Factor 7
Exposure Factor 8
Exposure Factor 9
Exposure Factor 10
Exposure Factor 11
Exposure Factor 12
Exposure Factor 13
Exposure Factor 14
Exposure Factor 15
Exposure Factor 16
Exposure Factor 17
Exposure Factor 18
Exposure Factor 19
Latent constructs describing exposure variables

Figure S2: Rotated factor pattern based on tetrachoric and polychoric, common exploratory factor analysis of measured exposure variables in the training dataset; limited to observed (measured) variables that did not have a standardized regression coefficient ≥ 0.5 for at least one latent construct.

Number of participants: 24,411

Standardized regression coefficient:

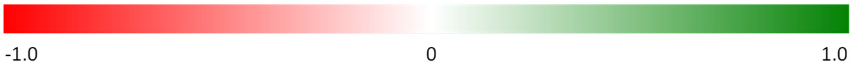

Supplement: Supplementary file 1 [file nutrients-13-01364-s001.zip › nutrients-1159535-supplementary.pdf]
